# Synthesis of Laboratory Ultrasound Contrast Agents

**DOI:** 10.3390/molecules181013078

**Published:** 2013-10-21

**Authors:** Jingam Park, Donghee Park, Unchul Shin, Sanghyub Moon, Chihyun Kim, Han Sung Kim, Hyunjin Park, Kiju Choi, Bong-Kwang Jung, Jaemin Oh, Jongbum Seo

**Affiliations:** 1 Department of Biomedical Engineering, Yonsei University, Maeji-ri, Heungeop-myeon, Wonju, Gangwon-do 220-710, Korea; E-Mails: sense@yonsei.ac.kr (J.P.); white6565@nate.com (D.P.); fdlnlmt@naver.com (U.S.); shnoom@naver.com (S.M.); chihyun@yonsei.ac.kr (C.K.); hanskim@yonsei.ac.kr (H.S.K.); 2 School of Electronic and Electrical Engineering Sungkyunkwan University, Suwon 440-746, Korea; E-Mail: hyunjinp@skku.edu; 3 Div. Respiratory Viruses, Center for Infectious Diseases, Korea National Institute of Health, Gangoe-myeon, Cheongwon-gun, Chungbok 363-700, Korea; E-Mail: cemovis@gmail.com; 4 Department of Parasitology and Tropical Medicine, Seoul National University College of Medicine, Seoul, Korea E-Mail: mulddang@gmail.com; 5 Department of Chemistry and Medical Chemistry, Yonsei University, Maeji-ri, Heungeop-myeon, Wonju, Gangwon-do 220-710, Korea; E-Mail: jaemin.oh@yonsei.ac.kr

**Keywords:** ultrasound contrast agent, microbubble, cavitation, synthesis of microbubble, size control

## Abstract

Ultrasound Contrast Agents (UCAs) were developed to maximize reflection contrast so that organs can be seen clearly in ultrasound imaging. UCAs increase the signal to noise ratio (SNR) by linear and non-linear mechanisms and thus help more accurately visualize the internal organs and blood vessels. However, the UCAs on the market are not only expensive, but are also not optimized for use in various therapeutic research applications such as ultrasound-aided drug delivery. The UCAs fabricated in this study utilize conventional lipid and albumin for shell formation and perfluorobutane as the internal gas. The shape and density of the UCA bubbles were verified by optical microscopy and Cryo SEM, and compared to those of the commercially available UCAs, Definity^®^ and Sonovue^®^. The size distribution and characteristics of the reflected signal were also analyzed using a particle size analyzer and ultrasound imaging equipment. Our experiments indicate that UCAs composed of spherical microbubbles, the majority of which were smaller than 1 um, were successfully synthesized. Microbubbles 10 um or larger were also identified when different shell characteristics and filters were used. These laboratory UCAs can be used for research in both diagnoses and therapies.

## 1. Introduction

Ultrasound contrast agents (UCAs) composed of engineered microbubbles, which ensure stability in the blood stream, have been available for more than ten years. The echogenicity of blood can be increased due to impedance mismatch and nonlinear oscillation [1,2,3,4,5,6,7]. Contrast between the blood and surrounding tissue in ultrasound imaging is enhanced by introducing UCAs into the bloodstream, and UCAs are widely used for diagnostic purposes in echocardiography [8,9,10,11,12,13]. UCAs have also been successfully used in oncology to increase visibility of breast, prostate, and liver tumors [14,15]. Lately, targeted UCAs have been created by attaching target-specific ligands to an UCA shell so that molecularly-specific ultrasound imaging has become plausible.

UCAs have also been widely used in research regarding targeted drug delivery, drug delivery through the blood brain barrier (BBB), and high-intensity focused ultrasound (HIFU) [16,17,18,19,20]. The nonlinear response of UCAs to the ultrasound field can cause localized microstreaming or jet streaming accompanied by cavitation, allowing for alterations in the permeability of the skin, cell membrane, and BBB without significant damage to normal tissue [21,22]. This effect can be used to facilitate drug delivery to a localized area [23,24,25,26]. Lately, targeted drug delivery has been made more plausible by attaching ligands to the UCA shell [18,19,27,28,29,30,31]. On the other hand, the use of UCAs during HIFU can reduce the energy required to damage cancerous tissue by increasing heat deposition or cavitation [32,33,34,35].

The increase in the applications for UCA has stimulated research and development in UCA synthesis in both industry and academia [36,37,38]. Currently, more than five commercial UCAs are available for clinical use, and more than 20 potential UCAs have been tested around the world. Targeted UCAs are also commercially available [39,40,41]. Additionally, a number of different ways to synthesize UCAs using various shell materials and internal gas have been published [42,43,44]. Recently, nano-paramagnetic iron particle and nano-carbon tubes have also been used to synthesize multifunctional UCAs [45,46]. Although commercial products and the results from references provide a certain level of access to UCAs and show novel approaches for multifunctional UCAs, the global use of UCAs is still limited to relatively few researchers due to regional regulations and high cost [47,48]. In addition, since the commercially-available UCAs are mostly designed for ultrasound imaging purposes, most of these cannot be further manipulated to be combined with target drugs for optimal use in drug delivery.

We investigated a simple method to synthesize two types of UCAs in a laboratory environment so that the size and inner structure can be controlled and then evaluated the properties of the proposed UCAs. The synthesized UCAs were analyzed by cryo-SEM and high resolution optical microscopy to confirm bubble formation. The size distribution and response to ultrasound imaging were quantitatively measured and compared to those of commercial UCAs. To ensure safety, the toxicity of the synthesized UCAs was analyzed using 293A cells. Finally, the stability of the synthesized UCAs was measured by ultrasound imaging over a two week period. Although these evaluations are focused on the verification of basic properties of the synthesized UCAs, the proposed method of synthesis may provide the possibility to encapsulate or tag various therapeutic drugs in UCAs according to aspects of their surface structure such as the presence of a lipid bilayer as well as properties such as hydrophobicity or hydrophilicity. Furthermore, the proposed UCAs could be further optimized for certain resonance frequencies by controlling their sizes.

## 2. Results and Discussion

### 2.1. Results

**Figure 1:**
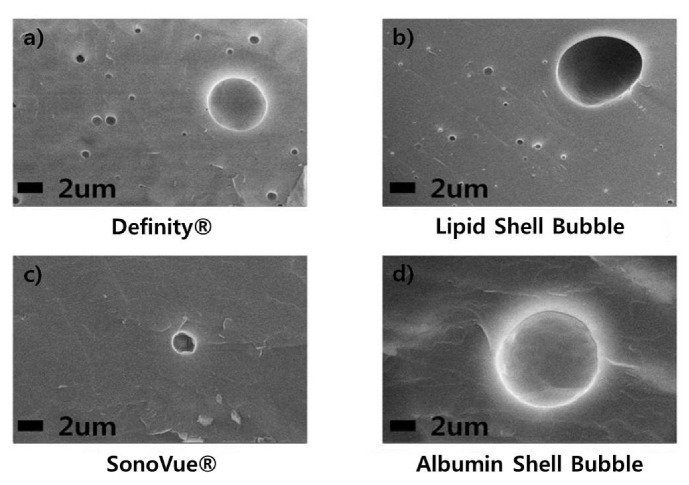
Cryo-SEM images of UCAs. Mirco-size circular bubbles can be observed in all four cases.

Cryo-SEM images show successful microbubble formation in the synthesized UCAs. As can be observed in Figure 1(a) and (b), the lipid shell UCA has a similar circular shape and size to Definity^®^ while the albumin shell UCA seems slightly larger than others on cryo-SEM. 

**Figure 2:**
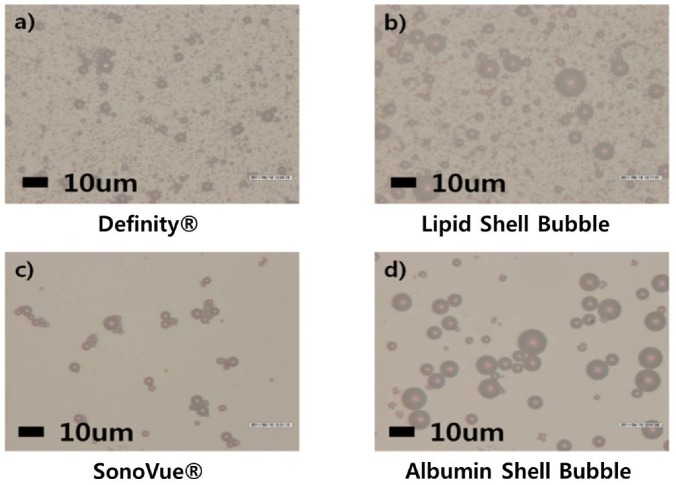
Optical images of the UCAs. The shape of the UCAs was observed under optical microscope at 1400× magnification.

The formation of microbubbles can also be visualized by optical microscopy, as can be seen in Figure 1 and Figure 2. Since optical microscopic images have moderate magnification compared to SEM, the overall density of the microbubbles in the individual UCAs can be qualitatively compared. As expected from the cryo-SEM images, the density of the lipid shell UCA is slightly less than that of Definity^®^. On the other hand, SonoVue^®^ has much lower microbubble density compared to Definity^®^ and appears similar to the synthesized albumin shell UCA.

**Figure 3:**
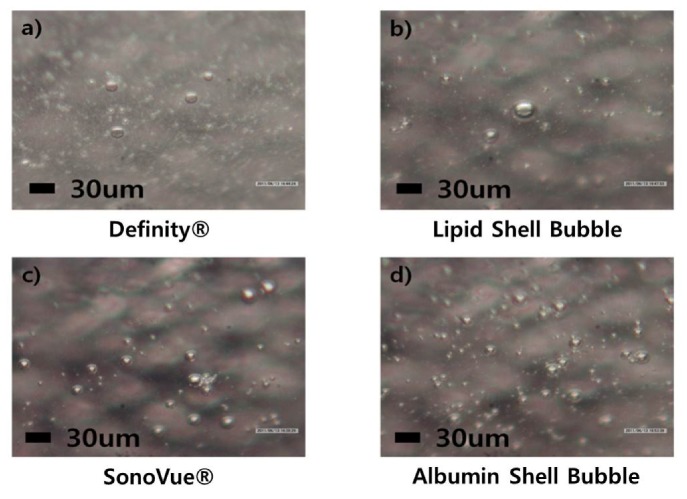
3D tomographic images of the UCAs. In order to confirm round shape microbubbles, 3D optical images were obtained at 400× magnification.

Figure 3 shows 3D tomographic images of individual UCAs at 400× magnification. Although only large bubbles can be visualized in 3D due to the image quality limit, spherical bubble formation can be observed clearly in all four UCAs.

**Figure 4:**
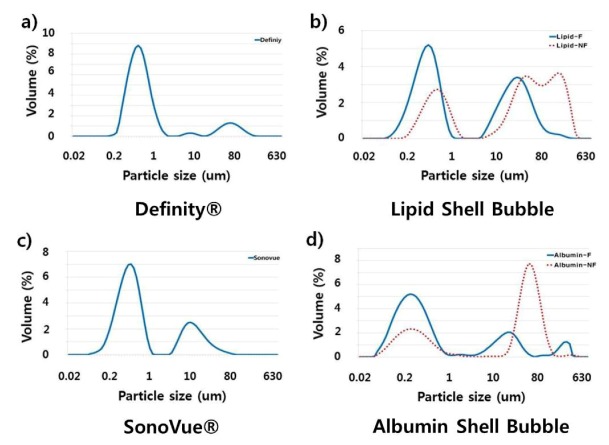
Size distributions of UCAs. The size distribution of UCAs was measured with a particle size analyzer (Mastersizer 2000, Malvern Instruments Ltd., UK) and plotted against volume. In this figure ‘NF’ indicated non-filtered data, and ‘F’ indicated filtered data, respectively. The results indicate that the synthesized UCAs have approximately five times more volume percent of microbubbles in the range of 10–100 μm compared to that of commercial UCAs. If the synsthesized UCAs was filtered once, the volume percent of the larger bubble was reduced approximately four times.

**Figure 5:**
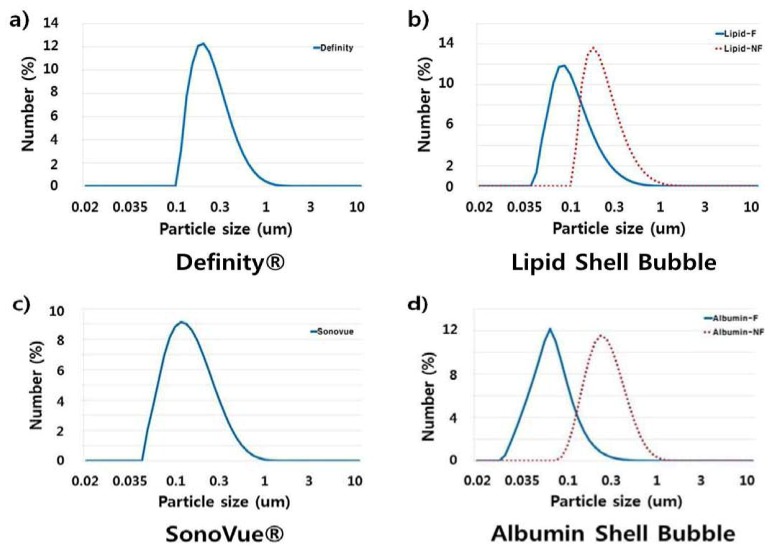
Size distributions of UCAs by the number of microbubbles. The majority of synthesized microbubbles are adequate to use as UCAs. The use of a 5 μm membrane filter seems to over-filter, resulting in small-sized microbubbles ranging from 1–3 μm.

The measured size distributions are shown in Figure 4 and Figure 5. As expected, commercial UCAs have a well-defined size distribution mainly between 0.05–1 μm. Synthesized UCAs also have large amount of bubbles in the range of 0.05–1 μm, but a relatively larger volume of tens of micrometers bubbles can be observed (Figure 4). 

If a 5 μm membrane filter was used to reduce the large bubbles, the portion of larger bubbles decreased significantly as can be seen in Figure 4. However, if the size distributions of bubbles were analyzed in terms of number instead of volume, the significant reduction of bubbles in the range of 0.1–1 μm can also be observed (see Figure 5). Hence, a membrane filter with larger pore size seems more adequate to obtain a bubble size more similar to that of commercial UCAs. 

**Figure 6:**
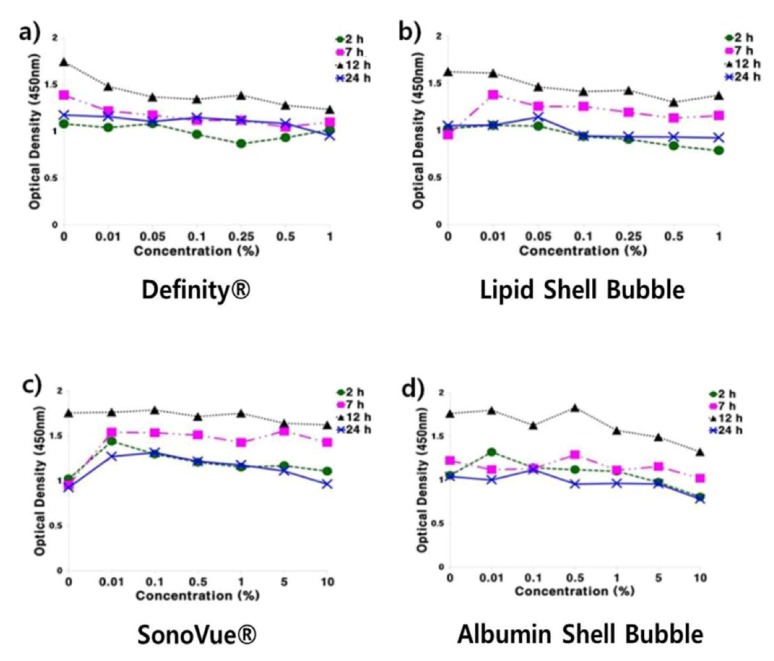
Cell toxicity test of the UCAs. The results indicate that all of the tested UCAs are not toxic to 293A human embryo kidney cell.

The MTT assay results are summarized in Figure 6. No significant changes in cell confluence can be detected in media containing up to 1% UCA in any experiment; hence, all the UCAs are non-toxic at the tested concentrations. Considering that the concentration used in the clinic is generally around 0.03%, the MTT assay results indicate that all of the UCAs are safe to use in animal experiments.

**Figure 7:**
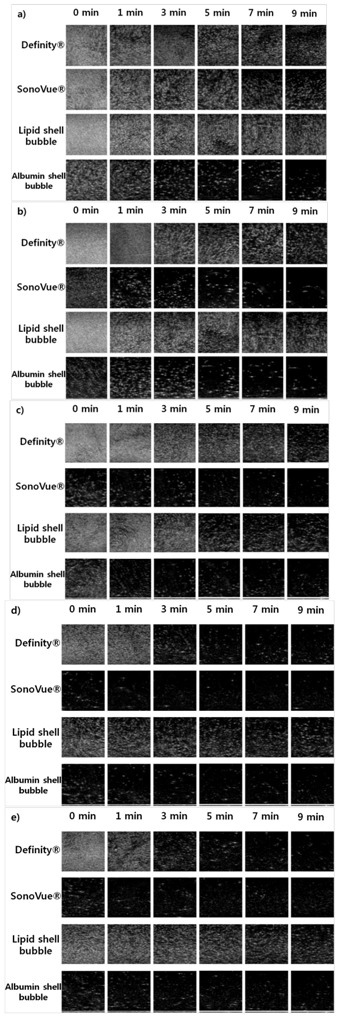
Ultrasound imaging comparison of two commercial UCAs and two synthesized UCAs over time (0, 1, 3, 5, 7, 9 min). (**a**) 3 days after synthesis. (**b**) 6 days after synthesis. (**c**) 9 days after synthesis. (**d**) 12 days after synthesis. (**e**) 15 days after synthesis. Ultrasound imaging results show that the synthesized microbubbles can be successfully used for imaging purposes. In particular, the synthesized lipid shell UCA shows almost equivalent imaging capabilities as Definity^®^. On the other hand, the synthesized albumin shell UCA seems to less effective in increasing ultrasound imaging contrast.

The characteristics of each UCA under ultrasound can be seen in Figure 7. As expected, the presence of a small amount of UCA increases the reflection so that the average brightness on 5 MHz ultrasound imaging increases to 160 dB in a water balloon. Additionally, the average brightness decreases exponentially with time due to the breakdown of microbubbles in the degassed water. Although the maximum points on the average brightness curve differ by up to 60 dB, the decay times of the microbubbles are equivalent. The main reason for the different maximum brightness may be the relatively small density of microbubbles in the SonoVue^®^ and albumin shell UCAs.

**Figure 8:**
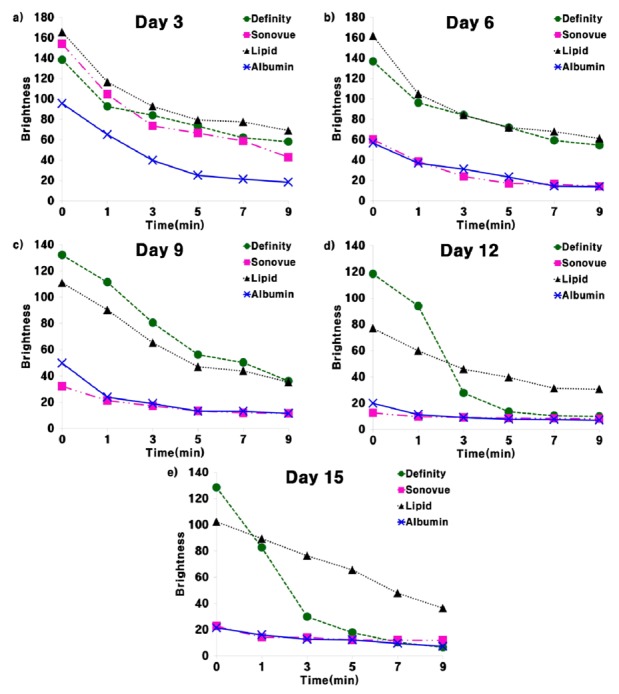
UCA lifespan. All the UCAs seem to have limited lifespan in the case of bolus injection. Additionally, the preservation period of all of the UCAs seems to be less than six days. SonoVue^®^ and albumin shelled UCAs seem to decay faster after opening the vial or synthesis.

Although UCAs are generally not intended to be used after the vial is opened, the long-term stability was evaluated to verify the lifespan of the synthesized UCAs. Figure 7 shows the durability of the average brightness over time (0, 1, 3, 5, 7, 9 min) after the opening of the commercial UCA vials or the synthesis of UCAs according to release date (6, 9, 12, 15 days after). The echogenicity of SonoVue^®^ and the albumin shell UCA show significant reductions after three days, suggesting that they should be used in experiments on the day of opening or synthesis (see Figure 7 and Figure 8). On the other hand, Definity^®^ and the lipid shell UCA show almost equivalent echogenicity values for the first three days (see Figure 7 and Figure 8b). However, the echogenicity decreased more than 20 dB after six days. This shows that Definity^®^ and the lipid shell UCA are slightly more stable after exposure to air but should be used in experiments less than three days after opening or synthesis. 

In these experiments, the formation of microbubbles was confirmed through optical and electronic microscopes, and the non-toxic nature of the synthesized UCAs was verified. Additionally, the possibility of size distribution control of the synthesized UCAs using a membrane filter was demonstrated. Although the need to preserve synthesized UCAs is inconvenient for later use, they have equivalent characteristics under ultrasound to commercial UCAs. Therefore, the proposed methods to synthesize UCAs could provide an alternative to commercial UCAs for laboratory experiments.

### 2.2. Discussion

In comparative observation of different UCAs with an optical microscope or a particle size analyzer, the dilution rate needs to be controlled according to the density of microbubbles. The dilution rate of lipid shell bubbles and Definity^®^ was set to 1 μL: 1 mL water, and that of the albumin shell bubble and SonoVue^®^ was set to 1 mL:20 mL due to the differences in bubble densities (Definity 1.2 × 10^10^/mL *vs.* SonoVue 2 2n10^8^/mL). Results indicates that albumin shell bubble and SonoVue^®^ still show lower density compared to lipid shell bubble and Definity^®^ even at 50 times higher dose (see Figure 2 and Figure 3). Albumin shell microbubbles is known to diffuse faster than lipid shell microbubbles [15,39], and albumin has a tendency to coagulate. If the albumin concentration exceeds a certain value, it has difficulty forming bubbles [15,39,49]. As expected, most of the generated albumin bubbles were observed to be attached to each other, as can be seen in Figure 2. Changes to the synthesis process such as adding phosphate and changing the solvent may be required for the generation of ideal albumin shell bubbles. On the other hand, lipid shell bubbles seem to form at the higher density without much of coagulation (see Figure 2). Additionally, the shell stiffness can be relatively easily controlled by adjusting the lipid type if lipid shell is used for microbubbles [50,51,52]. Since bubble response to ultrasound can be significantly affected by shell stiffness, as suggested by bubble models [49], further research needs to be conducted to determine the optimal lipids to use in the synthesis [50,53]. In addition, quantitative measures of shell elasticity with high precision equipment such as an Atomic Force Microscope (AFM) may be required. Evaluating shell elasticity could be helpful in designing better bubble dynamic models.

**Figure 9:**
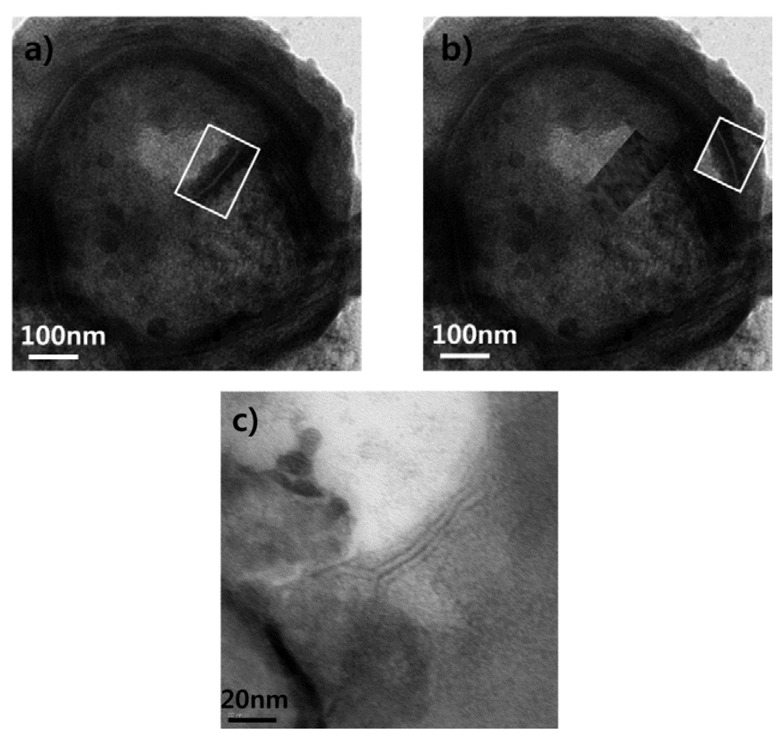
Staining lipid shell microbubble and the shell structure. Negative-stained TEM images with uranyl acetate and phosphotungstic acid was used to visualize the shell layer. The electron acceleration voltage was increased up to 120 kV to puncture the shell and to stain inside if possible. A fragment of the shell shown in Figure 9a was relocated to the shell surface to create Figure 9b. The fragment seems to fit the missing surface portion and form a complete shell surface. In other words, fragments of the shell do not seem to reform after being separated from the microbubbles. Figure 9c shows the bilayer-like structure of a fragment with a shell thickness of approximately 7 nm.

One of the merits of synthesizing microbubbles in the laboratory is that these bubbles can be combined with various drugs for use as a drug carrier [46,54]. In order to attach a drug molecule, the structure of the shell needs to be thoroughly characterized. In particular, the layer structure of the lipid-based microbubbles can limit the type of drug molecules which can be loaded inside or on the shell. Hence, the layer structure was visualized using negative-stained TEM images with uranyl acetate and phosphotungstic acid. Since staining can only affect the outer surface of the bubbles, the electron acceleration voltage was increased up to 120 kV. Accordingly, the synthesized mircobubbles can be punctured and both sides of lipid layer structure can be observed. As can be seen in Figure 9, the synthesized lipid shell shows a bilayer-like structure and the shell thickness is in the range of 5–10 nm. For this reason, the charged molecules seem to be encapsulated between these double layers. Further research will be conducted regarding the encapsulation of target molecules.

The layer structure of lipid shell bubble is generally considered as lipid monolayer [50,51,55]. However, the TEM images of the synthesized lipid shell bubble in Figure 9 show bilayer-like structure. This structure could be formed during the bubble rupture or the shell was formed bilayer-like during the synthesis. Considering the shell characteristics can be a critical issue on the drug loading location, the further clarification of shell structure is required using electron microscopy.

## 3. Experimental

### 3.1. Internal Gas: Perfluorobutane

**Figure 10:**
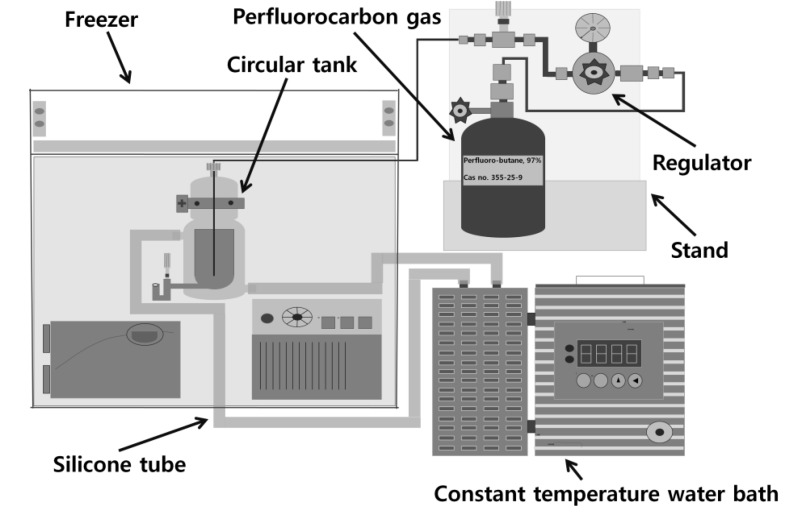
Low temperature workspace and liquefaction of perfluorobutane.

Perfluorocarbon gases are widely used as the internal gas in the synthesis of UCAs because these gases are inert and generally have low solubility in water [56,57,58]. Perflurobutane (Alpolo, FC-31-10, city, UK), in particular, was chosen for this laboratory synthesis because it can be easily liquefied and handled in a simple freezer due to its relatively high boiling temperature of −2.56 °C synthesis because iperfluorobutane, a low temperature workspace was created by modifying an ice cream showcase, as shown in Figure 10. Liquefied perfluorobutane was obtained through the outlet of the coolant circulation bath. Since the temperature of the manufactured workspace was approximately −15 °C and the shell material solutions were exposed to low temperatures for less than 15 min, solutions were not crystallized and could be directly used without lypophilization or rehydration.

### 3.2. Albumin Shell Bubble Synthesis

Since the amount of albumin is a critical factor in the formation of shelled microbubbles, different amounts of bovine serum albumin (Sigma, Aldrich, Saint Louis, MO, USA) were dissolved in 25 mL saline. The ratios between the albumin and saline mixture were approximately 1:2,500, 1:500, 1:250, 1:125, and 1:63 by weight. After the complete dissolution of albumin, 2 mL of the solution was transported to a vial and placed in the low temperature workspace for approximately 10 min. All the equipment including the micropipette, pipette tips, and vial caps were also cooled to prevent vaporization of liquefied perfluorobutane during handling. Approximately 20 μL of liquefied perfluorobutane was added into the solution and the vial was tightly sealed. Gas-filled albumin shell UCAs were synthesized by shaking the vials for 45 sec at 4,300 rpm with Vialmix^®^ (Lantheus Medical Imaging Inc., No. Billerica, MA, USA) at room temperature. The synthesized albumin shell UCA was filtered with a 5 UCAs were synthesized by shaking the vials for 45 secimately 20 mafoized UCA was preserved in a refrigerator at 5 °C for long-term stability testing. The stored albumin shell UCA was activated again with the Vialmix for 45 sec approximately 5 min before use.

### 3.3. Lipid Shell Bubble Synthesis

Saline, glycerol (Sigma Aldrich, Saint Louis, MO, USA), and propylene glycol (Acros Organics, Geel, Belgium, USA) were mixed in a ratio of 20:1:21 by volume. In 100 mL of the lipid saline solution, 0.1 g of 1,2-dipalmitoyl-sn-glycero-3-phosphocholine(DPPC) (Avanti Polar Lipids Inc., Alabaster, Alabama, USA) and 0.01 g of 1,2-dipalmitoyl-sn-glycero-3-phosphate (DPPA) (Avanti Polar Lipids Inc.) were added. The molar mass ratio of DPPC to DPPA was approximately 9:1. The solution was heated at 60 °C for 30 min on a hot plate to dissolve the DPPC and DPPA powders (PC-420D, Corning, Tewksbury, MA, USA). After complete dissolution, 2 mL of the solution was transferred to a vial and cooled in the manufactured workspace for approximately 10 min. The processes of formation, filtering, preservation, and activation of the gas-filled lipid shell UCA were identical to those of the gas-filled albumin shell UCA.

### 3.4. Analysis of UCAs

In the analysis, two synthesized UCAs and two locally available commercial UCAs were used. Definity^®^ and SonoVue^®^ were the two commercial UCAs used for comparative analysis with synthesized UCAs. These UCAs were simply chosen due to local availability.

The synthesized UCAs and two commercially available UCAs were activated, and approximately 10 μL of each UCA were loaded onto separate aluminum carriers for freeze fracture. All UCAs were cryogenically frozen in liquid nitrogen at −196 °C. The frozen samples were fractured with a freeze-fracture system (BAF060, BAL-TEC, Balzers, Los Angeles, CA, USA) at −120 °C, and the surface was coated with Pt ions at 1.5 kV and 60 mA. The processed samples were transported to an SEM (LEO 1455VP, Carl Zeiss, Hamburg, Germany) without exposure to air using a vacuum chamber transfer (VCT 100, BAL-TEC, Balzers). SEM images were obtained with an acceleration voltage of 15 kV at a stage temperature of −150 °C. 

Individual UCAs were also observed using an optical microscope (KH-770, Hirox, Tokyo, Japan). Approximately 10 μL of UCA were extracted from each vial and placed on slide glasses without dilution. The magnification for still images was 1400×, and 3D tomography was performed at 400× magnification. 

The size distributions of the UCAs were measured with a particle size analyzer (Mastersizer 2000, Malvern Instruments Ltd., Worcestershire, UK). The activated individual UCAs were added to deionized water in a ratio of 1:1000, respectively, and the diluted solutions were used for measurement. For each UCA, 15 repeated measurements were conducted to obtain a reliable particle size distribution.

**Figure 11:**
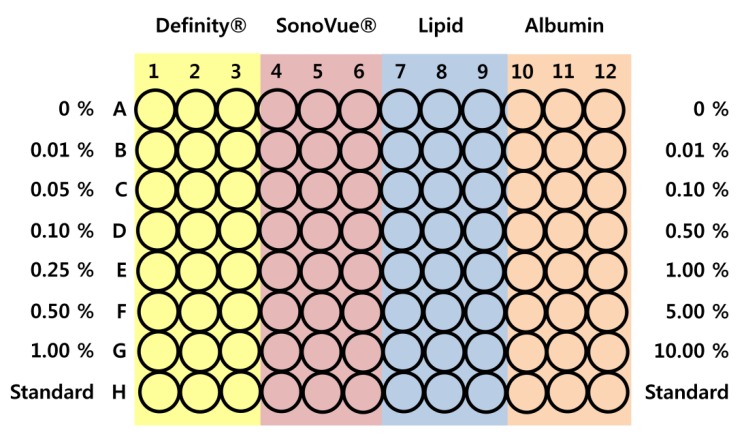
Toxicity evaluation setup. The 293A human embryo kidney cell line was used for toxicity testing.

Cell toxicity analysis was conducted using a cell counting kit-8 (CCK8) on cultured 293A human embryo kidney cell lines, which are widely used for DNA transfection experiments. 293A cells were chosen since this cell line can be easily cultured and the growth rate is relatively fast [59,60]. The 293A cells were cultured in 1.0 × 104 plates for 12 h. In each well, 10 μL of various concentrations of UCA were added and incubated for an appropriate time (2, 6, 12, and 24 h). After addition of 10 μL of CCK reagent to each well, the plate was incubated again for 1–4 h. The experimental conditions are illustrated in Figure 11.

**Figure 12:**
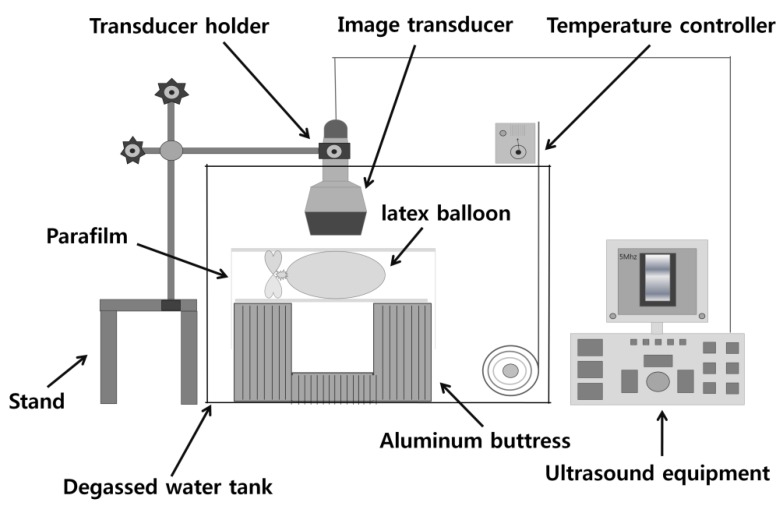
Experiment setup for UCA response to an ultrasound imaging system. All the prepared UCAs were imaged with a commercial ultrasound imaging system (Sonoace Pico^®^, Medison, Seoul, Korea).

The responses of the four UCAs to ultrasound were evaluated using a generic ultrasound imaging system (Sonoace Pico, Medison, Seoul, Korea). Figure 12 shows the experimental setup. Degassed water with an oxygen saturation of 50% or less was heated to 37 °C. Approximately 600 mL of degassed water was transported to a latex balloon, and individual UCAs were added in a ratio of 10,000:1. B mode images of the UCA solutions were obtained with a 5 MHz imaging probe at 0.59 MI output to prevent massive cavitation during image acquisition. In order to evaluate the lifespan of the UCAs in degassed water, a 3 cm × 3 cm region of interest (ROI) around the focus was chosen, and the average brightness of the ROI was measured every two minutes. To assess long-term stability, the lifespan test mentioned above was conducted every three days for a 15-day period after synthesis or the opening of the commercial UCA container.

## 4. Conclusions

Two different UCAs were synthesized in the laboratory and quantitatively evaluated using optical and ultrasonic devices. In addition, the toxicity of the synthesized UCAs was tested and compared to that of commercially-available UCAs. The comparison results indicate that the synthesized UCAs are adequate in size distribution and similarly respond to ultrasound as compared with commercial UCAs. The optical analysis of the synthesized lipid shell UCA shows the double layer shell structure. This information will help to determine the process of encapsulation of various drug molecules into UCAs. These UCAs could play a role as protection agents for drug molecule up to the delivery location and a role as physical delivery agents by proper ultrasound sonication. Although further improvements in longevity are required, we believe that synthesized UCAs can increase the general access to UCAs.
